# Evaluation of a multisite educational intervention to improve mobilization of older patients in hospital: protocol for mobilization of vulnerable elders in Ontario (MOVE ON)

**DOI:** 10.1186/1748-5908-8-76

**Published:** 2013-07-03

**Authors:** Barbara Liu, Ummukulthum Almaawiy, Julia E Moore, Wai-Hin Chan, Sharon E Straus

**Affiliations:** 1 Regional Geriatric Program of Toronto Sunnybrook Health Sciences Centre, 2075 Bayview Avenue, Toronto, ON M4N 3M5, Canada; 2 Li Ka Shing Knowledge Institute, St. Michael’s Hospital, 30 Bond Street, Toronto, ON M5B 1W8, Canada; 3 Department of Medicine, Faculty of Medicine, University of Toronto, 1 King’s College Circle, Medical Sciences Building, Toronto, ON M5S 1A8, Canada

**Keywords:** Mobilization, Frail, Older adult, Hospital, Quality improvement

## Abstract

**Background:**

Functional decline is a common adverse outcome of hospitalization in older people. Often, this decline is not related to the illness that precipitated admission, but to the process of care delivered in hospital. The association between immobility and adverse consequences is well established, yet older inpatients spend significant amounts of time supine in bed. We aim to implement and evaluate the impact of an evidence-based strategy to promote early mobilization and prevent functional decline in older patients admitted to university-affiliated acute care hospitals in Ontario, Canada. We will implement a multi-component educational intervention to support a change in practice to enhance mobilization of older patients.

**Methods/design:**

Implementation of our early mobilization strategy is guided by the Knowledge to Action Cycle. Through focus groups with frontline staff, we will identify barriers and facilitators to early mobilization. We will tailor the intervention at each site to the identified barriers and facilitators, focusing on the following key messages: to complete a mobility assessment and care plan within 24 hours of the decision to admit patients aged 65 years and older; to achieve mobilization at least 3 times per day; and, to ensure that mobilization is scaled and progressive. The primary outcome, number of patients observed out of bed, will be documented three times per day (in the morning, at lunch and in the afternoon), two days each week. This data collection will occur over 3 phases: pre-implementation (10 weeks), implementation (8 weeks), and post-implementation (20 weeks).

**Discussion:**

This is the first large, multisite study to evaluate the impact of a multi-component knowledge translation strategy on rates of mobilization of older patients in hospital. Our implementation is framed by the Knowledge to Action Cycle, and the intervention is being adapted to the local context. These unique features render our intervention approach more generalizable to multiple practice settings. Contextualization of the intervention has also facilitated engagement of participants from multiple hospitals. Upon completion of this study, we will better understand the barriers and facilitators to implementing an early mobilization strategy across a spectrum of hospitals, as well as the impact of a mobilization strategy.

## Background

Hospitalization can be a pivotal event in a senior’s life. Seniors have higher rates of adverse events, surgical complications, and nosocomial infections than younger people [1]. They are at risk for hospital-acquired delirium, increased length of stay, re-admission and loss of the capacity for independent living [1-3]. Over one third of older adults (aged 65 years and older) develop a new disability in an activity of daily living during hospitalization, and half of these individuals never recover that lost function [2,3].

Functional decline is common in older people who are admitted to hospital, and this has been found to be more a consequence of hospitalization than of their presenting illness [1,3-5]. For example, a study conducted on inpatient medicine units found that almost 20% of older inpatients (aged ≥65 years) who were independently ambulant two weeks prior to their admission required assistance at discharge [5]. The decline in functional ability that older patients experience in hospital may be related to extended periods of time spent supine in bed. Based on direct observation of patients admitted to general medical units, older patients who were previously ambulatory spent 83% of the day lying in bed [6]. Muscle strength is lost at a rate of up to 5% per week in young and older adults [7]. Immobility is also associated with qualitative changes in muscle protein and increased inflammatory markers [8-10]. Older adults often enter hospital with lower baseline muscle strength and mass than younger patients, and only a short time in bed is required to compromise an older person’s ability to ambulate independently.

Adoption of best practices to prevent hospital-acquired complications including functional decline in frail older patients can result in benefits in both patient and system-level outcomes, including decreased length of stay, decreased alternate level of care (ALC) days, and improved functional and cognitive status in older people. A Cochrane systematic review of multidisciplinary exercise interventions (where exercise was defined as any physical intervention program designed to maintain or improve a patient’s strength or functional status) for acutely hospitalized, older medical patients identified 9 trials that enrolled more than 4,000 patients [4]. Multidisciplinary exercise interventions were found to increase hospital discharge to home (number needed to treat 16 [95% confidence interval [CI] 11 to 43]), and to decrease hospital length of stay (weighted mean difference −1.2 days [95% CI −1.9 to −0.2]) and total hospital costs (weighted mean difference −279 [95% CI −492 to −65]) [4]. Indeed, the review found that with exercise, patients may go home one day earlier, and 6 more patients out of 100 may go home instead of to a care facility. These interventions may reduce hospital costs by $300 per patient per hospital stay [4]. An individual patient data meta-analysis identified that patients who require assistance or supervision to ambulate at admission are most likely to benefit from inpatient exercise [11]. Despite this available evidence, a recent survey of hospitals in Ontario, Canada, found that hospitals were unlikely to have a protocol or performance measure for prevention of functional decline in hospitalized elderly, highlighting a significant evidence to practice gap [12].

Evaluation of early mobilization strategies (defined as assessing patients for mobility and functional status within 24 hours of admission and encouraging appropriate activity immediately) has demonstrated the benefits in stroke patients [13], orthopedic patients [14,15], ventilated patients in the intensive care unit [16], and in patients with community-acquired pneumonia [17]. In randomized trials involving these patients, early mobilization has been shown to decrease acute care length of stay by 1.1 days [17]; shorten duration of delirium [16]; decrease risk of depression [13]; improve return to independent functional status; and increase rates of discharge to home [14,16]. Most studies described a strategy to encourage range of motion exercises in intubated patients or strategies to change from horizontal to upright position for at least 20 minutes within 24 hours of hospitalization and progressive movement with each subsequent day of hospitalization.

The objectives of the current study are to implement and evaluate the impact of an evidence-based strategy to promote early mobilization and prevent functional decline in older patients admitted to acute care academic facilities in Ontario, Canada. We will implement a multi-component educational intervention to support a change in practice related to mobilization of older patients. The mode of delivery of the education will be contextualized; however, the targeted change in practice will be consistent across all sites.

## Methods/design

### Setting

This study is being conducted in 14 of 15 eligible university-affiliated hospitals that provide acute hospital care for inpatients aged 65 and older in Ontario, Canada.

### Study design

The impact of the knowledge translation (KT) intervention will be evaluated using an interrupted time series design. In the 10-week pre-implementation period, we will engage local users and contextualize the implementation strategy. This period will be followed by an 8-week implementation roll-out period and then by a 20-week post-implementation period (Table 1).

**Table 1:** Duration of study phases and data collection

**Study phase**	**Pre intervention**	**Intervention**	**Post intervention**
Duration	Twice a week for 10 weeks	Twice a week for 8 weeks	Twice a week for 20 weeks
Primary Outcome Data points	20 audits	16 audits	40 audits
Secondary Outcomes	Chart abstractions of HDSRU	Chart abstractions of HDSRU	Chart abstractions of HDSRU

We are using an integrated KT approach, which refers to a collaborative process whereby researchers and research users work together to design the implementation process. This project was developed by knowledge users, specifically front line clinicians, patient advocates, and healthcare managers.

Our KT approach is informed by the Knowledge to Action (KTA) Cycle developed by Graham and colleagues [18]. It highlights processes relating to knowledge creation, distillation and use. In this model, the knowledge creation funnel represents knowledge generation, synthesis, and development of knowledge tools. The action parts of the cycle are based on a systematic review of planned action theories and include the processes needed to implement knowledge in healthcare settings, specifically, problem identification; assessment of determinants of KT; selecting, tailoring, implementing and evaluating KT interventions; and determining strategies for ensuring sustained knowledge use.

### Population

The target population will include patients aged 65 and older admitted to inpatient medicine units at an acute care or continuing care hospital. Patients designated as palliative will be excluded.

### Intervention

The implementation strategy is multi-component and will include a variety of components, tailored to the local context and including local champions, online and/or in-person educational interventions for healthcare providers and patients, printed education materials, implementation coaching from the central Mobilization Of Vulnerable Elders in Ontario (MOVE ON) team to participating sites, and an online community of practice. Prior to implementation of the early mobilization strategy on target units, relevant stakeholders and opinion leaders will be engaged at each hospital to ensure support for initiative. The targets for this engagement process include key stakeholders for the unit, such as patient care managers, directors, physicians, and nursing and allied health professional leaders. We will also recruit local working group members. These working groups are composed of staff at each participating hospital that will coordinate the implementation of the intervention. The working group will use information obtained from focus groups conducted with frontline staff to tailor the intervention components and adapt or create educational materials to suit the local context. The working groups will meet regularly until the end of the study. At each participating hospital, a local education coordinator, physician champion, and research coordinator will be identified to facilitate implementation and lead the local working group. Members of the central MOVE ON team (UA, JEM, and WC) will be designated as implementation coaches. Each participating site will be assigned to a MOVE ON implementation coach, who will provide support to the local implementation team.

Key to this initiative is the contextualization of the intervention at the local site. Team members will work with each hospital to identify local barriers and facilitators to implementation of the early mobilization strategy and to tailor the intervention to these factors. Instead of focusing on ensuring that the same intervention(s) is implemented at each site, the goal is to ensure that all sites commit to implementing the key messages that are: to complete a mobility assessment and mobility care plan within 24 hours of the decision to admit patients aged 65 years and older; to achieve mobilization at least three times per day; and, to ensure that mobilization is scaled up and progressive. Using this approach, each participating site tailors the intervention to their context to facilitate implementation and sustainability of early mobilization.

Development of our implementation strategy is based on the best available research evidence. We will conduct focus groups with frontline, interprofessional care staff (including physicians, nurses, physiotherapists, dieticians, pharmacists, occupational therapists, social workers, and case managers) from each of the targeted inpatient units. These one-hour sessions will be facilitated by a local champion and a research coordinator who has expertise in focus groups. We have used the work by Michie and colleagues [19] to inform the development of an interview guide for these focus groups that will be used to identify behavior change domains. Michie and colleagues identified 12 domains to explain behavior change, including knowledge; skills; social/professional identity; beliefs about capabilities; beliefs about consequences; motivation and goals; memory, attention and decision-making processes; environmental context and resources; social influences; emotional regulation; behavioral regulation; and the nature of the behavior. These domains will then be used to develop a tailored, multifaceted implementation intervention. We are interested in targeting behavior change for patients and healthcare providers, specifically to promote mobility of hospitalized older patients.

Four to eight healthcare providers will participate in each focus group. We anticipate completing one to three focus groups per unit. Sampling will continue until no new themes are identified. The focus groups will be digitally recorded, and the results will be transcribed and analyzed as described in the Qualitative Analysis section below. The results from this exercise will be used to map the intervention to the behavior change constructs [20]. For example, if the focus group identifies that lack of health care professionals’ knowledge of the impact of hospitalization on functional status of older people is a challenge, an educational intervention will be targeted as well as use of persuasive communication from a local opinion leader. If beliefs about consequences of mobilization (*i.e*., increasing the risk of falls) are identified as a challenge, information about the behavior outcome will be provided along with persuasive communication about the importance of mobilization of older people by an opinion leader. Similarly, if motivation is identified as a challenge, a social process for encouraging patient mobilization will be used.

In partnership with the local working groups and led by the local physician champion and education coordinator, we will tailor the implementation intervention based on the results of the focus groups and based on evidence from systematic reviews of the behavior change literature. Based on preliminary work to date with some of the hospitals, we have identified lack of knowledge of the consequences of hospitalization, beliefs about the consequences of mobilization, and lack of awareness of mobility strategies as important factors identified by healthcare providers. We therefore began developing a multifaceted, tailored implementation intervention targeting healthcare providers, patients and their family members. A total of 7 of 12 high quality systematic reviews of multifaceted interventions targeting healthcare professionals have identified that interventions combining two or more professional, organizational, financial, structural or regulatory interventions can improve healthcare professional behavior [21-32]. Tailored strategies are defined as strategies to improve professional practice that are planned, taking account of prospectively identified barriers to change. In their high quality systematic review, Baker and colleagues included 12 trials of tailored interventions in a meta-analysis and found tailoring to be effective [33].

Several enabling tools (*e.g.*, a mobility algorithm, care pathway, e-learning modules, printed education materials, order set, etc.) to support the early mobilization strategy have been developed by the central MOVE ON team based on preliminary results from focus groups and will be made available via an electronic portal. The portal will also be used to collect feedback from participating hospitals on their use of these tools and to share examples of how they were modified for their local context. This portal has been created to further develop this community of practice and as a site to share educational resources, tips on implementation, and local experiences across the collaborating hospitals. It will also include a step-by-step manual for education coordinators and research coordinators who are involved with this initiative. The tailored intervention will be implemented over an eight-week period. Four to eight weeks after the eight-week intervention period (during the 20-week post-implementation period), we will conduct semi-structured interviews with healthcare personnel, the local working group, and patients and/or their family members at each participating site. Exit interview participants will be recruited, on a volunteer basis, from among the front line clinical staff on the target units. Letters of invitation will be sent to patients and/or family members prior to discharge to ask if they want to be contacted for telephone interviews. We anticipate completing three to five staff interviews and three to five patient and/or family member interviews per site. The interviews will explore their perceptions of the intervention, the fidelity of the intervention, and suggestions for facilitating its sustainability. These interviews will be completed by a research coordinator from the central MOVE ON team and will be digitally recorded. The results will be transcribed; each interview will be assigned a unique identifier and analyzed as described in the Qualitative Analysis section below.

### Outcomes

Using an integrated KT approach, the outcomes were selected and prioritized by the team members (including healthcare providers, educators, patient advocates, and decision-makers) to ensure that they are patient-centred and relevant to the institutions. The outcomes that were selected will also be of interest to others interested in implementing this initiative in other provinces and jurisdictions.

The primary outcome is the percentage of ‘out of bed’ observations amongst older patients measured by an audit of directly observed activity at three points during the day. A negative default of lying in bed will be used if the information is not clear from direct observation (for example, if the patient is off the ward for a test). Through the intervention, we seek to decrease the number of patients observed to be in bed during the assessment periods. Precision and accuracy of this strategy for detecting mobilization events have been evaluated against observations conducted every 15 minutes (inter-rater agreement, kappa 0.83; positive likelihood ratio 12.2; negative likelihood ratio 0.06). Currently, our participating sites do not routinely collect data on mobilization of patients. Secondary outcomes include: acute length of stay, functional status at admission and at discharge (Activities of Daily Living [ADL]/ Instrumental Activities of Daily Living [IADL]), discharge destination, falls, injurious falls; and perceptions of patients, informal caregivers, family members, and healthcare providers (Table 2).

**Table 2:** Study outcomes and source of data

	**Outcome**	**Definition**	**Data source**
**Primary**	Frequency of mobilization of patient	% of patients documented ‘not in bed’ during audit	Direct observation
**Secondary**	Length of stay	Days	Chart abstraction or hospital decision support unit (HDSRU)
	Functional status at admission and at discharge (ADL/IADL)	% patients in each category of ADL status	Chart abstraction
	Discharge destination	% patients discharged to location other than LTC	Chart abstraction or HDSRU
	Falls	Number of incident reports filed	Chart abstraction or HDSRU
	Injurious falls (fractures, subdural hematoma)	Number reported	Chart abstraction or HDSRU
	Perceptions and satisfaction of patients, informal caregivers, family members and healthcare	Qualitative data	Interviews
	Rate of documentation	Change in documentation of baseline and discharge functional status	Document review

### Data collection

Once ethics approval is received at the participating sites, collection of pre-implementation data will begin. Data will be collected three times per day (in the morning, at lunch, and in the afternoon) and two days each week (excluding holidays and weekends). This data collection will occur over three phases: pre-implementation (10 weeks or 20 assessment days), implementation (8 weeks or 16 assessment days), and post-implementation (20 weeks or 40 assessment days) (Table 2). In order to detect a 10% decrease for the patients being in bed after the intervention, based on a power of 0.8 and type I error of 0.05, correlation for AR(1) of 0.4 and proportion of patients being in bed before the intervention at 66%, the required number of data collection points is 40.

Baseline data will be collected on eligible patients including age, gender, place of residence prior to admission, admitting diagnosis, and functional status prior to admission from the chart. This information will be collected retrospectively from chart review.

### Analysis

#### Quantitative analysis

The interrupted time series design will be used as it is more robust than a simple before-after study design. The primary outcome is the proportion of patients aged 65 and older who are mobilized three or more times daily during the audit. Mobilization is defined as not being in bed. The primary analysis will compare the proportion of inpatients aged 65 years and older who were mobilized in the pre-, during and post-intervention periods. We will also perform subgroup analyses across the different participating hospitals to provide site-specific data. The unit of experimentation will be based on multiple temporal assessments. For the primary outcome, 20 consecutive time points over 10 weeks will be used to create a pre-intervention model. To evaluate whether this pre-intervention model adequately describes the baseline, time series models will also be estimated for the entire 76 time points (pre- and post-intervention). We anticipate that each site will obtain data on 35 to 45 patients per audit. Segmented linear regression models will be performed to examine the impact of the intervention on the primary outcomes [34]. The initial time series regression model included terms estimating changes in the level and trend of outcome rates after intervention, in addition to the constant term, and the term estimating changes in baseline trend over time. Durbin’s alternative test [35] will be used to examine for the presence of serial autocorrelation among the observations, and models will be corrected for autocorrelation as required using the Cochrane-Orcutt methodology [36]. All statistical analyses will be conducted using SAS version 10.0, and statistical significance will be set at p <0.05.

#### Qualitative analysis

Content analysis of the transcripts will begin after the first completed focus group and will draw on grounded theory, although no theory will be developed [37].The goal of these interviews and the analysis of their content is to develop a synopsis of the understanding of the key domains of behavior change that need to be targeted on the various clinical units. Additional interview questions may be identified through this process; thus, post-implementation interviews will be modified as necessary. Analysis will be completed independently by two investigators. Written memos will be used to provide a record of the analytic process. The memos will capture the decisions and results of the analysis and help to develop propositions. Investigators will deliberately try to discount or disprove a conclusion drawn from the data. Reliability of the categories will be determined by the frequency or consistency with which they are indicated by participants in their interviews. All participants will be provided with a copy of the study report for their review and comments. Sampling will continue until no new themes are identified, and it is anticipated that approximately one to three focus groups will be conducted at each site. Analysis will be done using NVivo 10.

Content analysis of the staff and patient and/or family interviews will be done by two team members independently using the methods described above for the baseline focus groups. Sampling will be continued until no new themes are identified. It is anticipated that up to 10 interviews may be completed for each hospital.

### Study status

A launch meeting was held in 2012 with the participating teams in Toronto, Canada, and monthly phone calls are held for all participants to share experiences. All sites have completed their baseline data collection phase, and the tailored intervention is being rolled out at all sites. Approval has been received from the research ethics board at each of the participating hospitals.

## Discussion

This is the first large, multisite study to evaluate the impact of a multi-component KT strategy on rates of mobilization of older patients in hospital. Our implementation is framed by the KTA cycle [18], and the intervention is being adapted to the local context of the implementation site. The contextualization of the educational intervention will be informed by the focus group findings, which will reflect the unit characteristics, patient profile, staffing profile, and external environmental factors influencing the unit’s capacity for change. This unique feature renders our intervention approach more generalizable to multiple practice settings. It has also facilitated engagement of participants from multiple hospitals.

This study incorporates evidence-based success factors for quality improvement. First, we are ensuring that key stakeholders are engaged in all phases of the study. Our intervention is adapted to local context. Second, the education component of the intervention is reinforced through the use of enabling tools, coaching, and learning resources that are made available to all sites. Third, our intervention emphasizes the inclusion of all interprofessional team members to patient mobilization and does not rely on the contributions of a single individual or health profession [38].

There are several limitations to our study that should be acknowledged. First, the participating hospitals do not routinely collect data on mobilization of patients. Our primary outcome is based on three daily audits of patients being out of bed. Although we validated this measurement against a reference standard, it provides only an estimate of the total mobilization events that patients may undertake. We are not collecting data on evenings and weekends, when families tend to visit, since patients may be mobilized at a different rate during those times. One of the other goals of this project is to work with participating sites to develop a measure for assessing mobility that can be easily incorporated into paper and electronic medical records that are not too onerous for healthcare providers to use; its development will facilitate sustainability of this strategy. Second, the collection of outcome data (specifically, frequency of mobilization) by the research coordinator will not be blinded, and the research coordinator will be aware of the phase of implementation. We are, however, using standardized criteria for this outcome measure. Third, this project highlights the real world challenge of implementing evidence across multiple, different hospitals across a large province. There are always different initiatives underway at hospitals at the same time, all of which compete for hospital resources. We are vulnerable to the risk of initiative fatigue amongst frontline staff. We are using the local working groups to keep track of these other initiatives. Moreover, by allowing the local working groups to contextualize the mobilization intervention to their context, we anticipate alleviating some of this concern. Fourth, because this represents a real world implementation initiative, outcomes are subject to factors such as staff changes, infections outbreaks, and organizational restructuring. We will determine the impact of these confounders through our quantitative analysis as well as the qualitative component of our study.

Strengths of this initiative include the engagement of 93% of eligible academic institutions in Ontario, Canada, in this project. While inpatient medicine units are the initial targets of this strategy, efforts are already underway to scale this up across other units within the hospitals. Moreover, participating hospitals have expressed interest in using this provincial platform to implementing other strategies to optimize care of seniors in hospitals.

## Abbreviations

ADL: Activities of Daily Living; ALC: Alternate Level of Care; IADL: Instrumental Activities of Daily Living; KT: Knowledge Translation; KTA: Knowledge to Action Cycle; MOVE ON: Mobilization Of Vulnerable Elders in Ontario.

## Competing interests

SES is an associate editor of Implementation Science but was not involved with any decisions regarding this manuscript.

## Authors’ contributions

BL and SES conceived the study. BL and UA wrote the first draft of the paper from the original grant proposal, with all authors commenting on it and subsequent drafts. BL, UA, JEM, WC, and SES made substantial contributions to conception, design, and project management. DBF, JD, TI, CJ, SS, and MZ made substantial contributions to initial protocol development. UA, JEM, and WC provided coaching to all participating sites. All authors read and approved the final manuscript.

## Members of the MOVE ON Team

Baycrest Health Sciences: Sylvia Davidson, Terumi Izukawa (TI), Judy Ritchie, Shelly Ann Rampersad; Hamilton Health Sciences: Sharon Marr, Anne Pizzacalla, Sigrid Vinson; Health Sciences North: Andrea Lee, Christine Paquette, Debbie Szymanski, Padma Paranum; Kingston General Hospital: Richard Jewitt, Rachael Morkem, Johanna Murphy, Terry Richmond; London Health Sciences Centre: Trish Fitzpatrick, Karilyn Lamers, Alexander Nicodemo, Mary-Margaret Taabazuing; Hôpital Montfort: Caroline Borris, Émily Escaravage, John Joanisse, Philippe Marleau, Carolyn Welch; Mount Sinai Hospital: Natasha Bhesania, Selma Chaudhry, Rebecca Ramsden, Samir Sinha (SS), Tracy Smith-Carrier; North York General Hospital: Krysta Andrews, Gabriel Chan, Norma McCormick, Maria Monteiro, Kam Tong Yeung; The Ottawa Hospital: Frederic Beauchemin, Jan-Michael Charles, Lara Khoury, Barbara Power, Vicki Thomson; St. Joseph’s Healthcare Hamilton: Vincent G. DePaul, Diana Hatzoglou, Miranda Prince, Susan Ritchie, Kelly Turner, Bashir Versi; St. Michael’s Hospital: Wai-Hin Chan (WC), Rami Garg, Christy Johnson (CJ), Dorothy Knights, Christine Marquez, Julia E. Moore (JEM), Kasha Pyka, Lee Ringer, Sharon E. Straus (SES), Judy Tran, Maria Zorzitto (MZ); Sunnybrook Health Sciences Centre: Ummukulthum Almaawiy (UA), Deborah Brown-Farrell (DBF), Shima Deljoomanesh, Jocelyn Denomme (JD), Barbara Liu (BL), Beth O’Leary; Thunder Bay Regional Health Sciences Centre: Hanan ElSherif, Jennifer Hawley, Terry Robertshaw; University Health Network: Dunia Hamou, Mary Kay McCarthy, Andrew Milroy, Alistair Scott, Anne Vandeursen.
